# Peptidomics of an *in vitro* digested α-Gal carrying protein revealed IgE-reactive peptides

**DOI:** 10.1038/s41598-017-05355-4

**Published:** 2017-07-12

**Authors:** D. Apostolovic, M. Krstic, J. Mihailovic, M. Starkhammar, T. Cirkovic Velickovic, C. Hamsten, M. van Hage

**Affiliations:** 1 Department of Medicine Solna, Immunology and Allergy Unit, Karolinska Institutet and Karolinska University Hospital, Stockholm, Sweden; 2 Center of Excellence for Molecular Food Sciences, University of Belgrade, Faculty of Chemistry, Belgrade, Serbia; 3 Department of Internal Medicine, Södersjukhuset, Stockholm Sweden; 4 Ghent University Global Campus, Yeonsu-gu, Incheon South Korea; 5 Faculty of Bioscience Engineering, Ghent University, Ghent, Belgium

## Abstract

The mammalian carbohydrate galactose-α1,3-galactose (α-Gal) causes a novel form of food allergy, red meat allergy, where patients experience severe allergic reactions several hours after red meat consumption. Here we explored gastric digestion of α-Gal glycoproteins using an *in vitro* model. Bovine thyroglobulin (BTG), a typical α-Gal carrying glycoprotein, was digested with pepsin. The resulting peptides were characterised by SDS PAGE, immunoblot and ImmunoCAP using sera from 20 red meat allergic patients. During pepsinolysis of BTG, a wide range of peptide bands was observed of which 14 to 17 kDa peptides remained stable throughout the gastric phase. The presence of the α-Gal epitope on the obtained peptides was demonstrated by an anti-α-Gal antibody and IgE from red meat allergic patients. The α-Gal digests were able to inhibit up to 86% of IgE reactivity to BTG. Importantly, basophil activation test demonstrated that the allergenic activity of BTG was retained after digestion in all four tested patients. Mass spectrometry-based peptidomics revealed that these peptides represent mostly internal and C-terminal parts of the protein, where the most potent IgE-binding α-Gal residues were identified at Asn_1756_, Asn_1850_ and Asn_2231_. Thus allergenic α-Gal epitopes are stable to pepsinolysis, reinforcing their role as clinically relevant food allergens.

## Introduction

During the last decade a novel type of food allergy has been identified where patients report gastrointestinal symptoms, urticaria, angioedema, or anaphylaxis, not in the short time frame of typical IgE mediated allergy, but 3 to 6 hours after ingestion of mammalian meat such as beef, lamb or pork^1–7^. The reactions were shown to be caused by IgE antibodies directed against a carbohydrate epitope, galactose-α-1,3-galactose (α-Gal)^8^. Furthermore, a strong association with tick bites was revealed^9, 10^. This relationship was even further supported by the identification of the α-Gal epitope in the gastrointestinal tract of the European tick *Ixodes ricinus* ^11^. The number of diagnosed red meat allergy cases has increased over the past few years and have been reported worldwide^12^.

The α-Gal epitope is abundantly expressed on glycolipids and glycoproteins from non-primate mammals and some lower primates, but not in humans, where the gene encoding α-1,3-galactosyltransferase is not functional^13^. Although IgG antibodies to α-Gal are widely expressed in humans^14^, presumably in response to continuous exposure to the α-Gal epitope via gut microorganisms^15^, IgE antibodies to α-Gal are not.

Proteomics is an important tool in revealing and assessing the physicochemical properties of food allergens^16, 17^ and several proteomics studies have identified α-Gal-containing meat proteins that react with IgE from red meat-allergic patients^18–20^. Some of these proteins remain stable to thermal processing, showing that the allergenic properties are preserved in cooked meat^19, 20^. The long delay of symptoms in α-Gal allergy raises the question whether α-Gal glycoproteins have a retained IgE reactivity and allergenic properties when they appear in the circulation after digestion.

Many food allergens are stable to gastrointestinal (GI) digestion, which may contribute to their allergenicity^21, 22^. It has been postulated that food allergens must have properties that preserve their protein structure from degradation in the GI tract, such as resistance to low pH, bile salts and proteolysis^23–26^. Consequently, resistance to pepsin digestion in the stomach can be seen as a marker for potential allergenicity^21^. However some food allergens, e.g. allergens belonging to the PR-10 protein family, are less resistant to pepsin^22^ and furthermore even small peptides obtained after digestion of other food allergens can be allergenic^27^. Posttranslational modifications also have an important role in digestibility. For example, carbohydrate chains on the egg allergen ovomucoid contribute to increased resistance to proteolysis, and allergenic potency^28^.

Thyroglobulin is a dimeric 660 kDa glycoprotein with 13 N-glycosylation sites^29, 30^ endowed with three different glycan types (complex, hybrid oligosaccharides and high mannose type)^31^. Porcine thyroglobulin has been shown to contain complex glycans where the bi-antennary glycan harbours the galactose-α1,3-galactose-β1,4-N-acetylglucosamine epitope on one antenna and the galactose-β1,4-N-acetylglucosamine epitope on the second^32^. Bovine thyroglobulin (BTG) is heavily decorated with α-Gal and N-glycolylneuramic acid (neuramic acid or Neu5Gc) and is commonly used in the diagnosis of red meat allergy^10, 14^.

So far there is no knowledge whether the α-Gal epitope is part of complex and/or hybrid N-glycan types in red meat allergy and whether this has a potential impact on the delayed allergic response.

In order to reveal how α-Gal-containing glycoproteins are affected by gut exposure and if all types of α-Gal residues are IgE reactive and allergenic, we subjected BTG, as a model α-Gal-containing protein, to gastric digestion and investigated the obtained glycopeptides by immunoblotting, mass spectrometry and basophil activation assays.

## Results

### Pepsin digestion revealed hydrolyzed α-Gal-containing peptides

**Figure 1:**
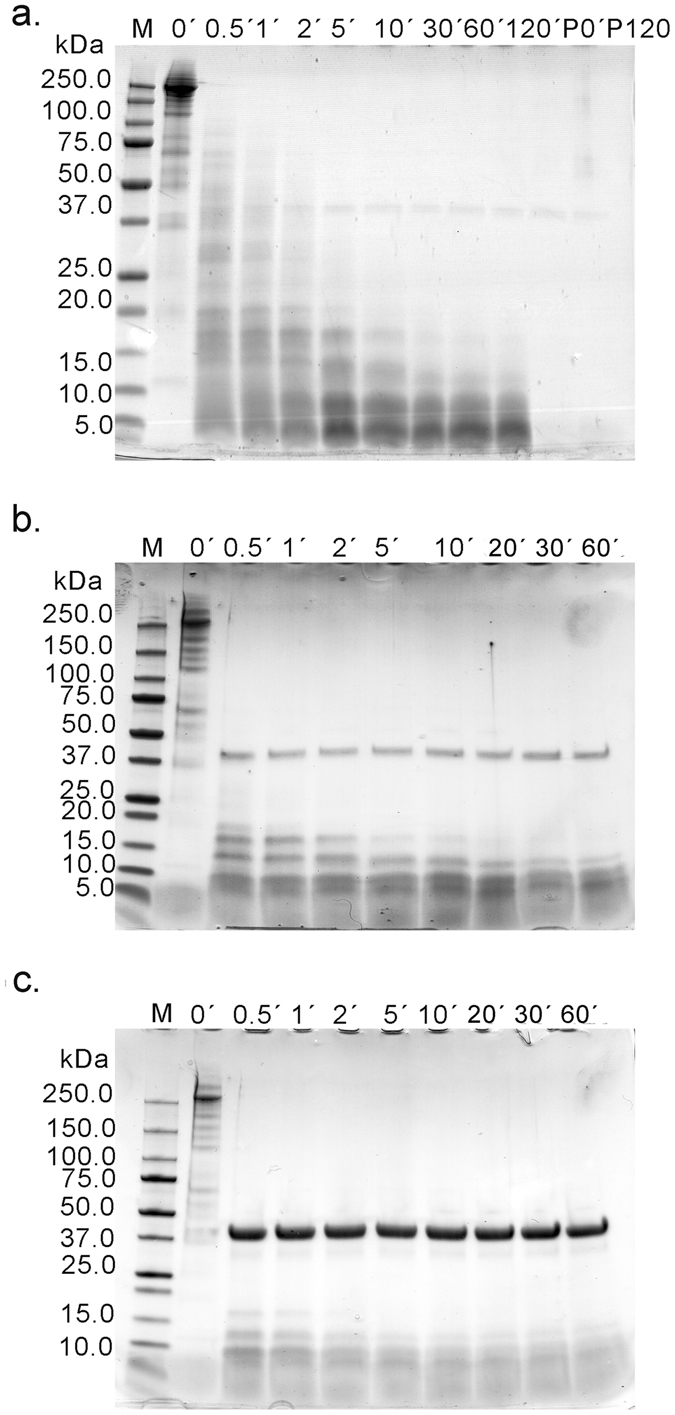
SDS PAGE analysis of gastric digestion of the α-Gal containing protein bovine thyroglobulin. (**a**) 0.2 U of pepsin per µg of protein; (**b**) 1 U of pepsin per µg of protein; (**c**) 10 U pepsin per µg of protein. Numbers above each lane represent digestion time in minutes. P0’ and P120’ represent a control solution with pepsin only at 0 and 120 minutes.

**Figure 2:**
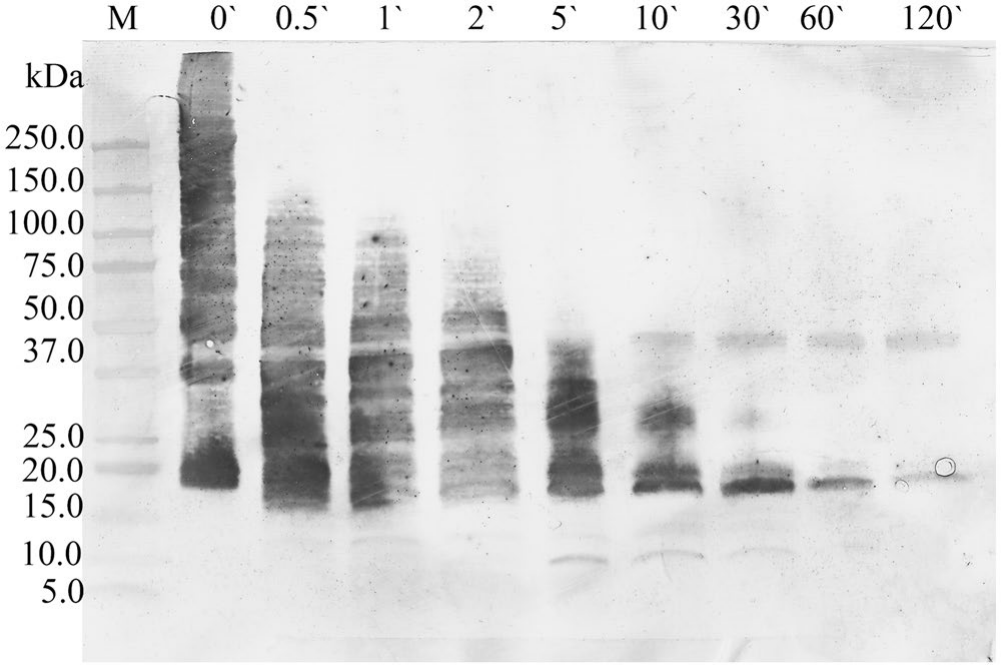
Anti-α-Gal-binding profile of gastric digestion of the bovine thyroglobulin under the physiological conditions with 0.2 U per µg of pepsin. Numbers above each lane represent digestion time in minutes.

Electrophoretic analysis of BTG under reducing conditions showed a major band at 270 kDa and lower molecular weight protein bands that represent products caused by reduction of disulfide bridges or impurities from other bovine proteins. BTG degraded quickly in the presence of 0.2 U of pepsin per µg of protein during *in vitro* digestion (Fig. 1a) and the degradation pattern was similar with or without the presence of phosphatidyl choline (PtdCho) vesicles (data not shown). After 30 seconds of digestion the major protein band disappeared and a wide range of peptide bands at molecular sizes of 100, 75, 50 and 40 kDa could be observed until 10 min of pepsinolysis. At 10 minutes, peptides with approximate molecular sizes of 15 kDa appeared and remained stable during 60 minutes of digestion (Fig. 1a). By increasing the concentration of pepsin 5- or 50-fold, representing the concentration used by US Pharmacopeia, the pepsinolysis progressed faster. Peptides of 15 kDa were present from 2 min of digestion up to 30 minutes for 1 U/µg of protein (Fig. 1b) or only for 2 minutes for 10 U/µg of protein (Fig. 1c). Peptides <15 kDa were present during the whole pepsinolysis. Gastric digestion of deglycosylated BTG could not be performed due to protein insolubility at acidic pH conditions (data not shown). The α-Gal-content of digestion products obtained under physiological conditions was visualized using immunoblot and a monoclonal anti-α-Gal antibody (Fig. 2). The result showed that α-Gal was present during the whole pepsinolysis and that final peptides obtained after 120 minutes still contained α-Gal. A weak α-Gal-binding was observed for pepsin (of porcine origine) at 37 kDa.

### α-Gal-containing peptides bound IgE from red meat-allergic patients

**Figure 3:**
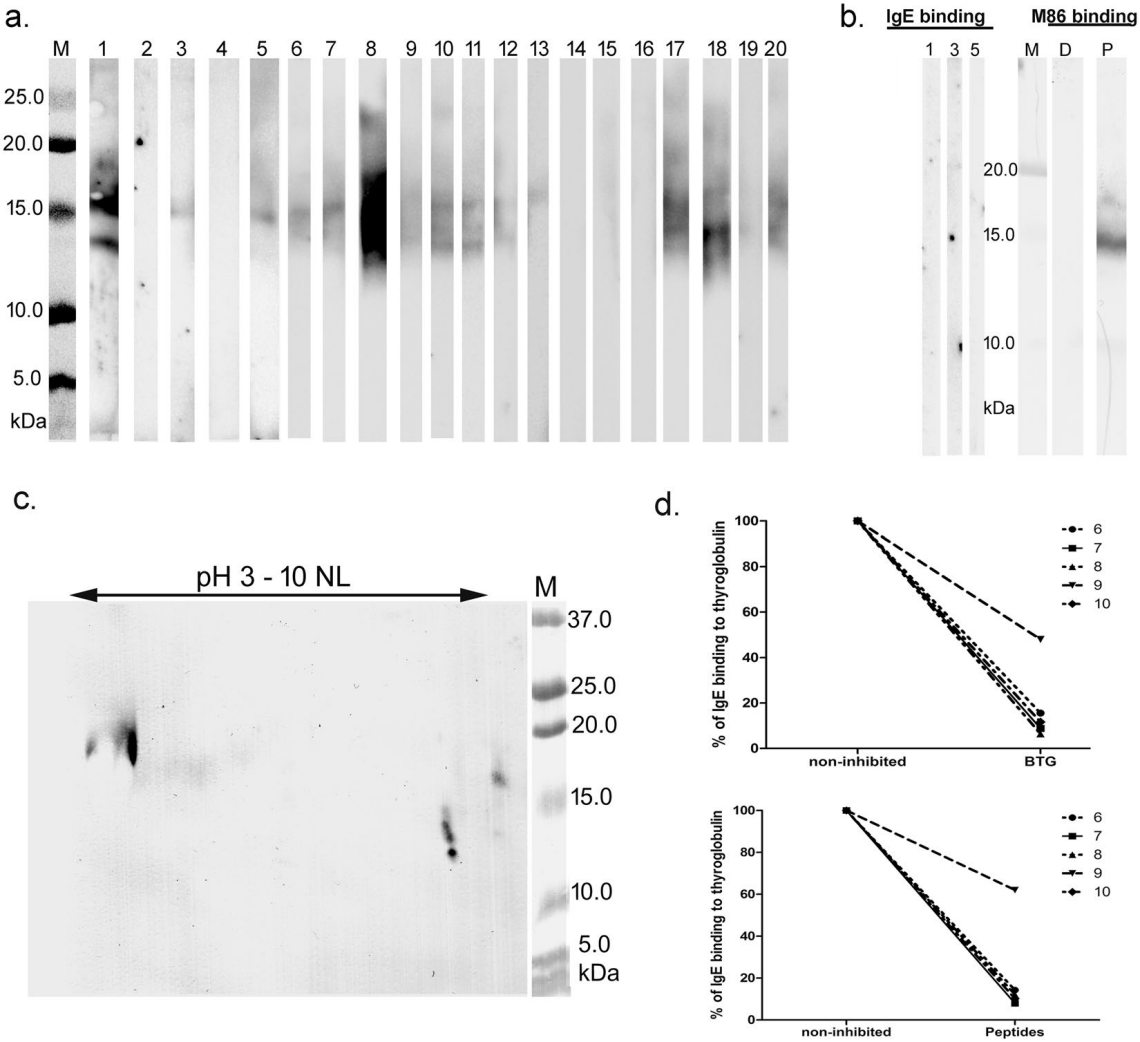
Allergenic properties of bovine thyroglobulin pepsinolysis products. (**a**) Individual patients’ IgE-binding properties on immunoblot with bovine thyroglobulin peptides obtained after 60 min of gastric digestion with 0.2 U per µg of pepsin. (**b**) Left side of the panel: IgE binding of three individual patients on deglycosylated peptides; right side of the panel: anti-α-Gal binding on α-Gal peptides-P, and deglycosylated peptides -D; M-Molecular weight markers. (**c**) 2D immunoblot of α-Gal peptides developed with the serum pool from 20 red meat-allergic patients. M-Molecular weight markers; (**d**) Inhibition of IgE binding to bovine thyroglobulin using preincubation with bovine thyroglobulin or thyroglobulin peptides obtained after 60 min of gastric digestion.

The IgE-binding properties of the peptides at different time points of digestion were visualized by immunoblot and a pool of sera from 5 red meat-allergic patients (Supplementary Figure S1). The results showed that α-Gal containing peptides bound IgE throughout the pepsinolysis, but the overall IgE reactivity decreased as proteins were digested. The glycopeptides obtained after 1 h of pepsinolysis where further separated on a 16% acrylamide gel to obtain better resolution of small molecular masses, transferred on a membrane and IgE-binding was evaluated using 20 individual sera with different IgE levels to α-Gal (median 23 kU_A_/l, range 6.3–100 kU_A_/l, Supplementary Table S1) (Fig. 3a). Fourteen out of 20 patient sera showed IgE-binding to the obtained α-Gal peptides in 14–17 kDa mass range. The IgE levels to α-Gal among the six sera lacking IgE binding to glycopeptides were below the median range (#2, 11 kU_A_/l; #4, 6.4 kU_A_/l; #14, 19 kU_A_/l; #15, 16 kU_A_/l; #16, 22 kU_A_/l; #19, 10 kU_A_/l).

To confirm that the observed binding was α-Gal specific, the peptides were deglycosylated with PNGase F and three individual sera were reanalysed (Fig. 3b). No IgE binding was observed and the absence of α-Gal was further confirmed with a monoclonal anti-α-Gal antibody (Fig. 3b). Further analysis using a 2D immunoblot developed with the serum pool (Fig. 3c) revealed that there are several spots representing peptides at 14 kDa with pI values in basic region, and several peptides at 17 kDa with pIs in both acidic and basic regions of the 2D immunoblot. IgE from red meat-allergic patients’ sera did not recognize peptides with molecular mass lower than 10 kDa.

In order to determine the proportion of α-Gal specific IgE antibodies that were directed against the peptides, adsorption experiments were performed using five individual sera. Each serum was pre-incubated with the peptides or undigested BTG as reference and the remaining IgE level to BTG was determined using ImmunoCAP. The results showed that these peptides were able to inhibit up to 86% of IgE-binding to α-Gal in four out of five patients. Homologous inhibition using undigested BTG revealed a similar pattern as the peptides (Fig. 3d).

### α-Gal-containing peptides retained the allergenic activity

**Figure 4:**
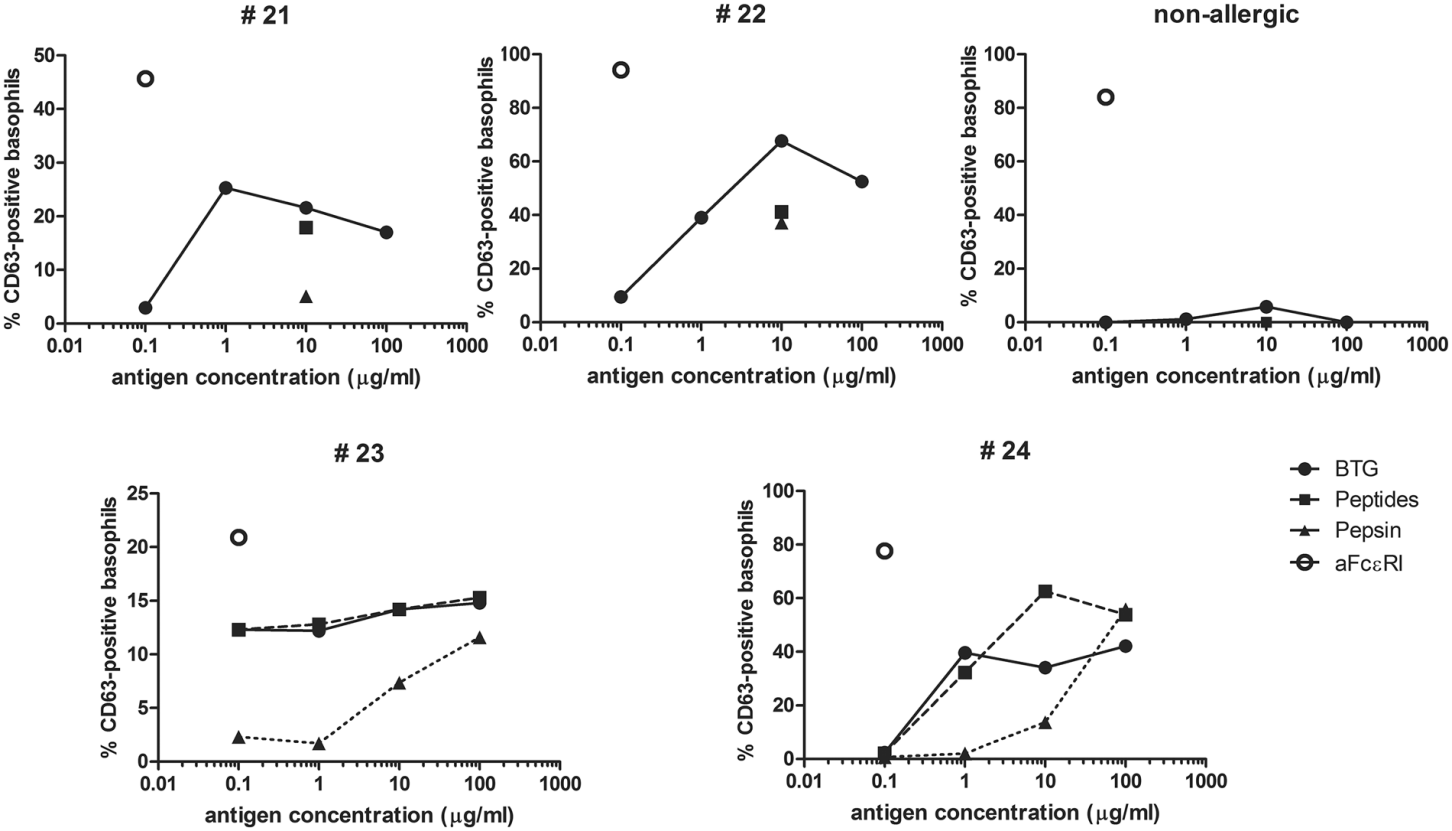
Allergenic activity of α-Gal IgE-reactive peptides. Allergenic activity of α-Gal IgE-reactive peptides, BTG, pepsin and anti-FcεRI (positive stimulation control) was determined by basophil activation in blood from four red meat-allergic patients and one non-allergic individual. Degranulation is presented as proportion (%) of CD63-positive out of CD203c-positive cells by flow cytometry (y-axes) in response to different allergen concentrations (x-axes).

The allergenic activity of α-Gal-containing peptides and BTG was investigated by basophil activation test using blood from four red meat-allergic patients (Table S1, #21–24) and one non-allergic individual. Pepsin was used as control antigen in an amount equal to the pepsin content of the peptide mixture. α-Gal-containing peptides induced basophil degranulation in a similar manner as BTG (Fig. 4). For patients #21 and #22 a single concentration of peptides or pepsin was tested (10 µg/ml). The allergenic activity for patient #23 was comparably lower, but still gave clearly positive results. In patients #22, #23 and #24 activation of basophils by pepsin was observed. None of the antigens activated basophils in the non-allergic individual, indicating that the observed reactions are IgE-dependent.

### Peptidomics of gastric peptides identified the most IgE reactive α-Gal residues

**Figure 5:**
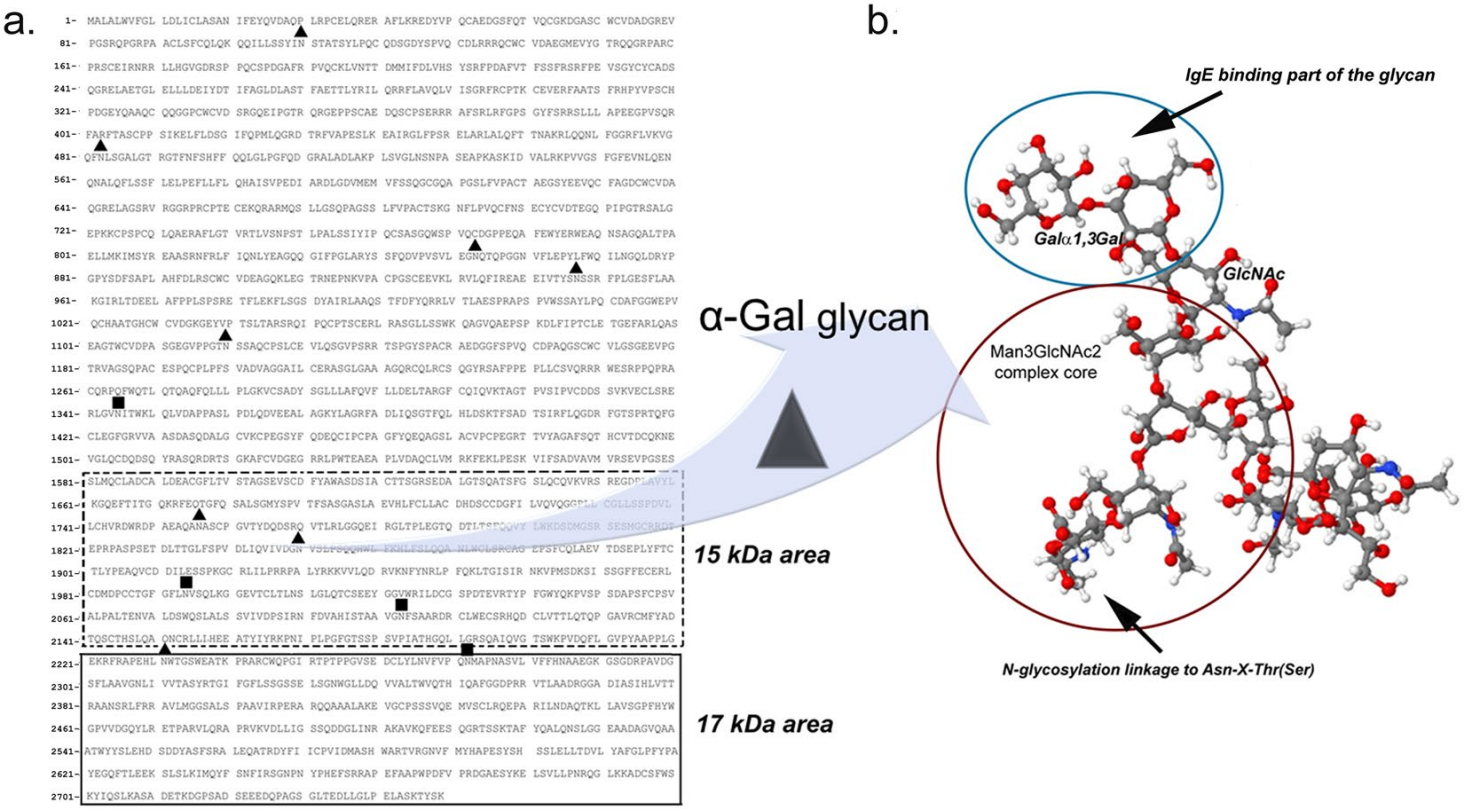
Peptidomics of α-Gal IgE-reactive peptides. (**a**) IgE reactive gastric peptides were identified using peptidomics and their corresponding regions in the bovine thyroglobulin sequence were identified (uniprot.org accession number P1027). Gastric peptides of 15–20 kDa, dark box; gastric peptides of 10–15 kDa, dashed box. Dark triangles above the sequence represent N-glycosylation sites with complex type of glycans; Dark squares above the sequence represent N-glycosylation sites with high mannose type of glycan. (**b**) Model of α-Gal glycan carried on peptide or protein. The IgE binding part of the glycan, the Gal-α1,3-Gal epitope, is marked in the blue circle; The complex Man_3_GlcNAc_2_ core of the glycan, where the end of the core is attached to protein/peptide with N-glycosylation linkage to Asn-X-Thr(Ser) sequence (black arrow), is marked in the red circle. Model was created using Avogadro software v1.1.1.

Peptides obtained after 60 minutes of pepsinolysis were separated on a 16% acrylamide gel and mass regions were analyzed by mass spectrometry (5–10, 10–15 and 15–20 kDa). The tryptic peptides identified by MS/MS analysis of the IgE reactive mass ranges (10–15 and 15–20 kDa; Fig. 3) obtained after 60 min of pepsin digestion are displayed in Supplementary Table S2. The 5–10 kDa mass range was not IgE reactive in immunoblot and the corresponding tryptic peptides were thus used to identify regions with low IgE reactivity (data not shown). The BTG sequence outlined with known N-glycosylation sites (high mannose type, squares above the amino acid sequence; complex type, triangles above amino acid sequence) and identified IgE binding regions is shown in Fig. 5. These regions were determined using mapped peptides from the IgE reactive mass ranges only, with corresponding theoretical pI values in line with experimental data as shown by 2D immunoblot in Fig. 3c.

The 17 kDa peptide from the pepsinolysis was mapped to the C-terminal region that contains two glycosylation sites (Fig. 5 box dark line), one highly mannose (square at Asn_2272_) and one complex type of glycan (triangle at Asn_2231_). Since highly mannose type glycans do not contain α-Gal residues, the 17 kDa peptide can thus only contain one α-Gal glycan (at Asn_2231_). Peptides excised from the bands of 10–15 kDa could be mapped over the whole sequence of BTG. However, an internal region of the protein sequence specific for peptide sizes between 10–15 kDa could be identified (Arg_1626_ until Asn_2169_, Fig. 4 box dotted line). This region contains two complex type glycans, at Asn_1756_ and Asn_1850._ Thus, the possibility of more than one α-Gal containing peptide at around 15 kDa cannot be excluded.

**Figure 6:**
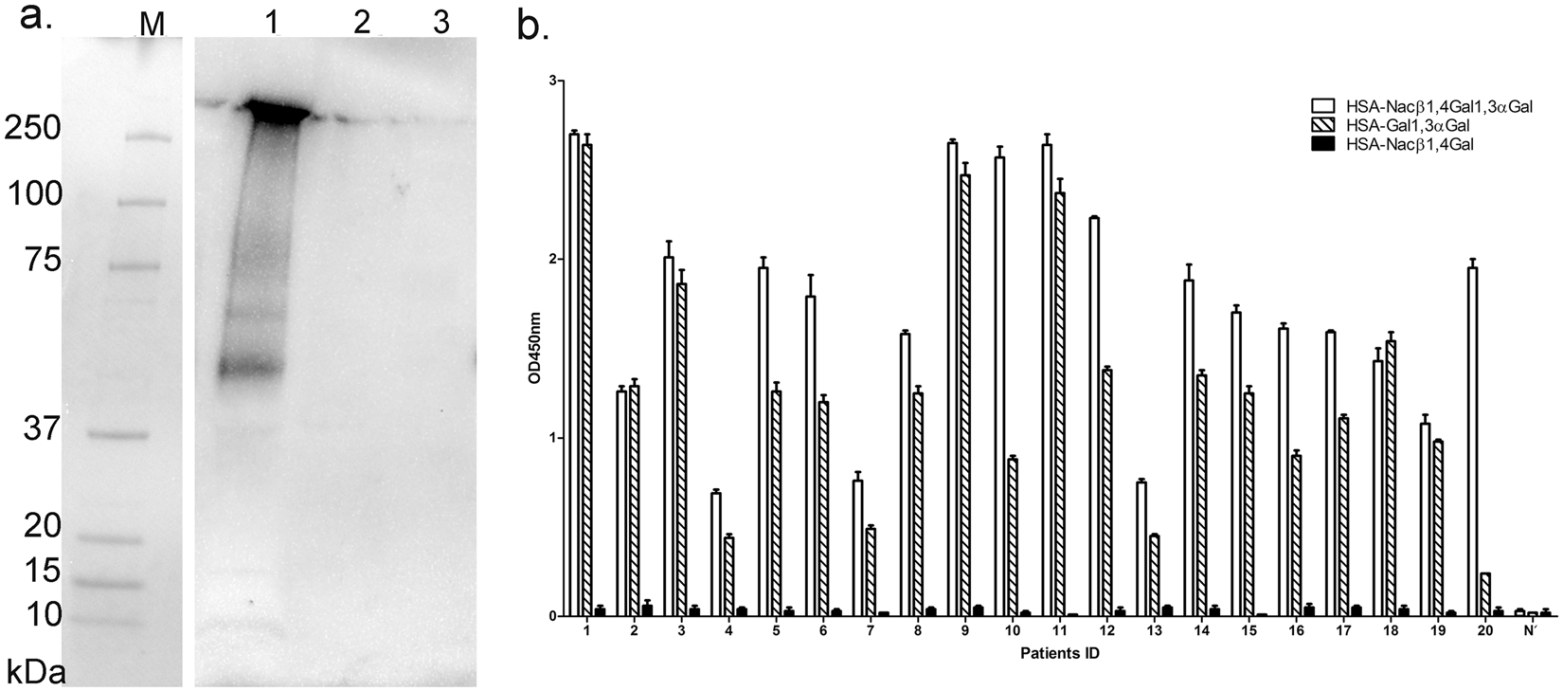
Mapping and influence of other glycans on IgE-reactive peptides. (**a**) Detection of Neu5Gc on bovine thyroglobulin and the corresponding digestion products using an anti-Neu5Gc antibody. Lane 1–Bovine thyroglobulin; Lane 2–Bovine thyroglobulin peptides obtained after 60 minutes of gastric digestion; Lane 3–Porcine pepsin only as control after 60 minutes of digestion; M–Molecular weight markers. (**b**) Patient IgE binding to HSA-NAc-β-1,4-galactose-α-1,3-galactose, HSA-galactose-α-1,3-galactose and HSA-NAc-β-1,4-galactose as measured by ELISA.

In order to explore whether these α-Gal containing glycopeptides had hybrid or complex N-glycans, an immunoblot of BTG and the digested peptides was probed with an anti-Neu5Gc antibody. The presence of Neu5Gc was confirmed on intact BTG (lane 1 Fig. 6a) but not on the pepsinolytic α-Gal containing peptides (lane 2, Fig. 6a).

As the corresponding complex glycans in porcine BTG contain the galactose-α1,3-galactose-β1,4-N-acetylglucosamine and galactose-β1,4-N-acetylglucosamine structures, the IgE binding against HSA-NAc-β-1,4-galactose, HSA- NAc-β-1,4-galactose-α-1,3-galactose (α-Gal trisaccharide), HSA- galactose-α-1,3-galactose (α-Gal disaccharide) and HSA was evaluated by direct ELISA. All 20 red meat-allergic sera tested showed strong IgE binding against the α-Gal trisaccharide and α-Gal disaccharide (Fig. 6b). No IgE binding to N-acetyl-β-1,4-galactose nor HSA (data not shown) was observed.

## Discussion

α-Gal rich foods elicit delayed allergic symptoms in red meat-allergic patients and thus the digestive processes in the gut are likely a prerequisite for the allergic responses. Here we explored the effect of gastric digestion of the α-Gal carrying glycoprotein BTG and investigated the allergenic properties of the pepsinolytic peptides. α-Gal has shown to be a clinically relevant food allergen and evaluating its stability to digestion is an important part of assessing the allergenic risk of this novel food allergen as GI stability and resistance to pepsin digestion directly affect the allergenicity.

We show that α-Gal glycopeptides obtained during *in vitro* digestion of BTG using a traditional pepsin digestibility model^33^ retained their IgE potency throughout pepsinolysis. When we evaluated the impact of the gastric phase, applying the digestibility assay by *Moreno* and co-workers^34^, the degradation pattern of BTG was similar with or without PtdCho vesicles, where 10 minutes of pepsin digestion generated peptides at 14–17 kDa that were observed throughout the simulated gastric phase. The presence and stability of the α-Gal epitope on peptides during the whole process of pepsinolysis was verified using an anti-α-Gal monoclonal antibody.

Even if the glycan is still attached to the protein backbone, the IgE-binding properties can be changed due to e.g. loss of protein conformation or due to digestion influencing the presentation of α-Gal residues. By using a serum pool from five red meat-allergic patients we showed that their IgE repertoire was able to bind peptides obtained after 60 minutes of pepsinolysis. Furthermore, we noticed that the majority of patient sera bound peptides of approximately 14 and 17 kDa. However, no clear relationship between binding intensity and the IgE level to α-Gal was noted. The IgE binding was strongly diminished when the glycan was removed from the peptides, pointing to the fact that the glycan is the sole target of red meat allergic patients’ IgE response. On the 2D immunoblot these glycopeptides showed to have acidic and basic pI values, which is different from the 4.5 pI value of the parent protein. We observed that a high proportion of the IgE reactivity to BTG in red meat allergic patient sera was diminished by preincubation with the pepsinolytic glycopeptides, as demonstrated by more than 80% inhibition of IgE responses to BTG in 4 out 5 sera tested. The last serum reached only 32% of inhibition, which is probably due to its high IgE level to α-Gal. All sera showed the same pattern in homologous inhibition with BTG, which suggests that the overall α-Gal IgE binding capacity of BTG is retained throughout digestion. Thus the observed incomplete inhibitions are probably due to excess of IgE and/or low affinity of IgE rather than epitope availability. More importantly, the positive basophil activation for all four tested patients demonstrated that the BTG glycopeptides retained their allergenic activity. In one patient we observed a general low but positive activation with patterns comparable to other patients. In some patients, activation was also observed against higher concentrations of pepsin. This is probably due to the porcine source of the enzyme that as such was shown to be α-Gal containing (seen as a weak band at 37 kDa when using the monoclonal anti-α-Gal antibody, Fig. 2).

Our peptidomics approach revealed that the obtained IgE reactive BTG glycopeptides mainly represent internal and C-terminal parts and do no not cover all available α-Gal glycans. Comparing our peptidomics data with previously identified glycosylation sites, the 17 kDa peptide contained one possible α-Gal glycosylation site only. Since the patients reacted to this peptide, we could pinpoint an IgE reactive α-Gal residue at this site close to the C-termini of BTG^31, 35^. Furthermore, a region with two potential α-Gal residues at Asn_1756_ and/or Asn_1850_ was identified solely in IgE reactive peptides at 10–15 kDa. This region does not seem to contain Neu5Gc. In BTG, this complex glycan seems to comprise the most allergenic α-Gal epitopes since all patients tested had the strongest IgE-reactivity to these peptides. As galactose-β1,4-N-acetylglucosamine is a part of the complex BTG glycan, we used direct ELISA to conclude that red meat-allergic patients’ IgE did not bind galactose-β1,4-N-acetylglucosamine itself and that there was a slight difference in IgE binding to α-Gal di- and trisaccharides. We have previously shown that red meat allergic patients have a selective IgE response to the α-Gal glycan that does not involve the protein backbone and, that Neu5Gc is not the target of the IgE response^36^. Plum and colleagues have shown that the IgE binding is localized to both galactose molecules but the the galactose-α1,3-galactose-β1,4-N-acetylglucosamine was not included in their study^37^. The only known cross-reactivity of the α-Gal IgE response was reported by Rispens and co-workers who showed that IgE from red meat-allergic patients could bind to the blood group B antigen, which is structurally similar to α-Gal^38^.

While the IgE reactive peptides had a size of 14 and 17 kDa, resembling small proteins capable of entering the intestinal mucosa for further enzymatic processing, the smaller peptides of up to 10 kDa were not recognized by IgE. Nonetheless, α-Gal residues will be presented to the gut immune system bound to a carrier (peptide or lipid), which together with the food matrix and other cofactors will influence the digestive process and subsequent IgE-mediated reactions. Our results showed rapid digestion into smaller peptides, hence a possible fast appearance of α-Gal in the circulation may be possible which is in contrast to the delayed appearance of symptoms *in vivo*. Here one must be aware that *in vitro* pepsinolysis assays only mimic initial parts of the very complex processes in human gastrointestinal tract. Furthermore, a meat meal contains other non-protein components which can influence the enzyme activity and delay uptake of peptides. In line with this, some patients report stronger reactions to fatty meats suggesting that α-Gal glycolipids and/or lipoprotein aggregates are of importance for further processing and uptake. However, such results have not yet been published. Nonetheless, retained IgE reactivity after gastric digestion is a crucial first step in investigating the allergenicity of α-Gal containing food allergens.

In conclusion, we show that α-Gal peptides are present during the whole gastric phase of BTG digestion. The most IgE reactive peptides were shown to contain up to two α-Gal epitopes per peptide and they are probably responsible for the retained allergenic activity of the peptide mix. The obtained peptidomics insight assists in understanding the role of α-Gal as a clinically relevant food allergen.

## Methods

### Patient material

Serum or heparinized blood from 24 patients reporting delayed allergic reactions following consumption of red meat and attending the Allergy Unit at Södersjukhuset, Stockholm, were enrolled in the study. All patients had been examined by a physician experienced in allergic diseases and were diagnosed with red meat allergy.

### Ethics Statement

The study was approved by the local ethics committee of Karolinska Institutet (2014/847-32 and 2016/1447-32) and all experiments were in accordance with relevant guidelines and regulations. Sample collection was done after written informed consent from the study participants.

### Chemicals

Bovine thyroglobulin (BTG), porcine pepsin (P-6887) and trypsin from Porcine Pancreas (Proteomic Grade, BioReagent, Dimethylated) were obtained from Sigma-Aldrich (St. Louis, MO, USA). HRP-conjugated mouse anti-human IgE (B3102E8, Abcam, UK), goat anti-human IgE (KPL, Gaithersburg, MD, USA), monoclonal mouse anti-α-Gal (M86, Enzo Life Science Inc, Farmingdale, NY, USA), AP-conjugated goat anti-mouse IgM (Southern Biotech, Birmingham, AL, USA) as well as FITC conjugated anti-CD63 and PE conjugated anti-CD203c (clones CLBGran/12 and 97A6, respectively, Immunotech, Marseille, France) antibodies were purchased. PNGase F kit for deglycosylation was obtained from New England BioLabs (Ispwich, MA, USA). HSA-NAc-β-1,4-galactose, HSA-NAc-β-1,4-galactose-α-1,3-galactose and HSA- galactose-α-1,3-galactose were obtained from Dextra Laboratories, Reading, UK. Deionized water for all experiments was purified in a Milli-Q system (Millipore, Molsheim, France). All other reagents (analytical grade) were purchased from Sigma-Aldrich.

### *In vitro* gastric digestion

*In vitro* gastric digestion was performed as previously described^34, 39^. BTG was chosen as a model α-Gal-containing protein and deglycosylated thyroglobulin was obtained using enzymatic deglycosylation^36^. Pepsin activity was verified according to manufacturer’s instructions prior to experiments. Gastric digestion was performed with or without the presence of phosphatidyl choline vesicles made from dry Egg l-PtdCho by dispersion in warm SGF and sonication at 5 °C (10 min set at 30% power, 9⁄10 power cycle). BTG was dissolved in simulated gastric fluid (SGF: 0.15 M NaCl, adjusted pH 2.5 with 1 M HCl) at a concentration of 5.55 mg/ml. The solution was mixed with PtdCho vesicles (1:1.2, v⁄v) and the pH was adjusted to 2.5 with 0.3 M HCl if necessary. After incubation at 37 °C for 15 min, a solution of pepsin 0.32% (w⁄v in SGF) was added at an approximately physiological ratio of enzyme⁄substrate (1:20, w⁄w), i.e. 182 U pepsin per mg of test protein. The digestion process was performed at 37 °C and aliquots were withdrawn from a single mixture of digestion at different time points up to 120 min for further analysis. The digestion was stopped by adding 200 mM ammonium bicarbonate, raising the pH to 7.5. Additional digestions were performed at 1U and 10U ﻿of pepsin﻿ per µg of protein for comparative purposes.

### SDS PAGE analysis

Digestion products were analyzed by SDS PAGE under reducing conditions. The analysis was carried using a BioRad MiniProtean II cell system and BioRad TGX gradient precast gels (any kDa or 4–20%, BioRad Laboratories, Hercules, CA, USA) or 16% acrylamide hand-cast gels.

### Immunoblot analysis

Following SDS-PAGE, digests were transferred to PVDF membranes (0.2 µm pore size) using a BioRad turbo system. Membranes were then washed by MiliQ H_2_O and for IgE-binding analysis, 1% HSA in PBS-T (containing 0.05% Tween) was used as a blocking solution, while for α-Gal binding 1% BSA in PBS-T. Incubation was performed at room temperature (RT) for 1 h. After blocking, the membrane was incubated overnight with individual sera from α-Gal allergic patients (patients #1–15 in Supplementary Table S1) diluted 1:4, or a serum pool of 5 allergic patients (patients #1–5 in Supplementary Table S1). Following wash, detection was carried out with HRP-conjugated mouse anti-human IgE (1:2500 dilution) and development with chemiluminescence substrate for HRP detection on ChemiDoc system (BioRad). Three individual sera (#1, #3 and #5 in Supplementary Table S1), were also tested on peptides which were subjected to the enzymatic deglycosylation.

Additionally, a 2D immunoblot with a serum pool made from all 20 sera was developed as previously described^40^. For α-Gal detection, the membrane was incubated for 3 h at RT with the anti-α-Gal antibody (diluted 1:3) followed by alkaline phosphatase (AP) conjugated goat anti-mouse IgM antibody (diluted 1:3000) for 1 h at RT. Visualization for AP was performed using nitro blue tetrazolium (NBT) and 5-bromo-4-chloro-3-indolyl phosphate (BCIP) substrates (BioRad). For detection of Neu5Gc, BTG and peptides obtained after 60 minutes of pepsinolysis as well as pepsin only were separated on 15% acrylamide gel, transferred onto PVDF membranes blocked with 1% HSA in PBS-T for 3 h at RT. Detection of Neu5Gc was preformed using chicken IgY anti-Neu5Gc antibody (BioLegend, San Diego, CA, USA, dilution 1:1000) overnight at 4 °C and donkey anti-chicken IgY labelled with HRP (Thermo Fischer Scientific, Rockford, IL, USA, dilution 1:7500) for 1 h at RT.

### Inhibition experiments

The capacity of α-Gal peptides to inhibit IgE binding to solid phase-bound α-Gal was measured using the ImmunoCAP System (Thermo Fisher Scientific, Uppsala, Sweden). Serum from 5 red meat-allergic patients (Patients #6–10 in Supplementary Table S1) was pre-incubated with BTG or α-Gal peptides obtained after 1 h of gastric phase before measurement of IgE using the bovine thyroglobulin ImmunoCAP.

### Basophil activation test

Allergen-specific basophil degranulation was analyzed by monitoring the basophil activation markers CD203c and CD63 as previously described^41^. Heparinized venous blood samples from four red meat-allergic patients (IgE range 5.2–>100 kU_A_/l against α-Gal, see Table S1) ﻿and one non-aller﻿gic individual were analyzed with BTG, α-Gal peptides obtained after 1 h of gastric phase and pepsin from porcine mucosa as control. Ten-fold serial dilutions of antigen were added to blood and incubated for 30 minutes at 37 °C. The pepsin amount was set up to be equal as in the peptide mixture. Anti-FcεRI (Bühlmann Laboratories AG, Schönenbuch, Switzerland) was used as positive control. The samples were further incubated with FITC conjugated anti-CD63 and PE conjugated anti-CD203c monoclonal antibodies, lysed and analyzed by flow cytometry using a BD FACS Canto II (BD Biosciences, San Jose, CA) and data were analyzed using FlowJo (Treestar, Ashland, OR). Basophils were identified by gating at least 300 CD203c-positive cells and the magnitude of allergen-activation was calculated and expressed as the percentage of CD63-positive cells among the gated basophils.

### Peptidomics analysis

The samples obtained after 60 minutes of pepsinolysis were run on 16% acrylamide gel. Bands in mass range of 5–10, 10–15 and 15–20 kDa were subjected to in gel digestion and MS analysis on EASY nanoLC II system coupled with LTQ Orbitrap XL (Thermo Fisher Scientific Inc., Bremen, Germany), previously calibrated with the ProteoMass^™^ LTQ/FT-Hybrid ESI Positive Mode Cal Mix (MSCAL5 SUPELCO, Sigma-Aldrich) as described previously^19^. The experiments were performed in duplicate.

### Identification of peptides containing α-Gal residues

Peptide identification was based on MS/MS data analysis in Proteome Discoverer 1.3.0339 (Thermo Fisher Scientific Inc., Bremen, Germany). Search was performed against BTG FASTA database, with SEQUEST algorithm. Two missed trypsin cleavages were allowed per peptide. Peptide tolerance and MS/MS tolerance were set to 10 ppm and 0.8 Da, respectively. Carbamidomethylation was set as static modification. False Discovery Rate was set to 0.01 for strict and 0.05 for relaxed mode of peptide discovery. The N-glycosylation sites were mapped based on information from uniprot.org (UniProtKB-P01267). Peptide calculator (http://pepcalc.com/) was used for theoretical mass calculation of obtained peptides and Peptide Cutter at ExPASy^42^ to map the theoretical pepsin cleavage sites (at Phe, Tyr, Trp and Leu) in the BTG primary structure for comparison to obtained peptides.

### Direct ELISA

The IgE binding of 20 red meat-allergic patients’ sera against HSA-NAc-β-1,4-galactose, HSA-NAc-β-1,4-galactose-α-1,3-galactose, HSA- galactose-α-1,3-galactose and HSA was analysed by direct ELISA. Half-area microtiter plates (96 wells, Greiner bio-one, Frickenhausen, Germany) were coated with 0.1 µg of corresponding antigen overnight at 4 °C. After blocking with 1% HSA in PBS-T, plates were incubated with sera from 20 red meat-allergic patients and 2 healthy controls (dilution 1:50) for 2 h at RT. Bound IgE was detected by using goat anti-human IgE conjugated to horseradish peroxidase for 1 h at RT and 3,3′,5,5′-tetramethylbenzidine (TMB) was added as substrate. The absorbance was measured at 450 nm.

## Electronic supplementary material

Supplementary information
