# Supplementary material for: Peptidomics of an in vitro digested α-Gal carrying protein revealed IgE-reactive peptides
Source: Sci Rep. 2017 Jul 12;7:5201. doi: 10.1038/s41598-017-05355-4 (PMC5507865; doi:10.1038/s41598-017-05355-4)
Supplement: Supplementary file 1 — Supplementary information [file 41598_2017_5355_MOESM1_ESM.pdf]

Supplementary information

Peptidomics of an *in vitro* digested a-Gal carrying protein revealed IgE-reactive peptides

D. Apostolovic[1], M. Krstic[1,2], J. Mihailovic[2], M. Starkhammar[3], T. Cirkovic Velickovic[2,4,5], C. Hamsten[1*], M. van Hage[1*]

[1]Department of Medicine Solna, Immunology and Allergy Unit, Karolinska Institutet and Karolinska University Hospital, Stockholm, Sweden;

[2]Center of Excellence for Molecular Food Sciences, University of Belgrade, Faculty of Chemistry, Belgrade, Serbia;

[3]Department of Internal Medicine, Södersjukhuset, Stockholm, Sweden

[4]Ghent University Global Campus, Yeonsu-gu, Incheon, South Korea

[5]Faculty of Bioscience Engineering, Ghent University, Ghent, Belgium

* these authors contributed equally

Correspondence

Marianne van Hage, MD, PhD

Karolinska Institutet

Department of Medicine Solna

Immunology and Allergy Unit

Karolinska University Hospital Solna L2:04

SE - 171 76 Stockholm, Sweden

Tel +46-8-5177 5942, Fax +46-8-33 57 24

E-mail: marianne.van.hage@ki.se

Figure S1. IgE binding profile of gastric digestion of the bovine thyroglobulin under the physiological conditions.

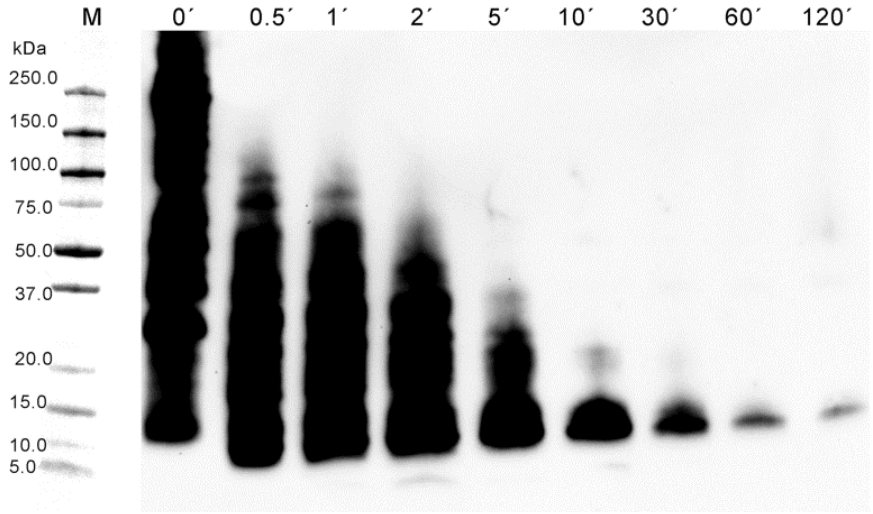

Tables

Table S1. Serological characteristics of red meat-allergic patients

No	Total IgE (kU$_A$/l)	Beef IgE (kU$_A$/l)	α-Gal IgE (kU$_A$/l)
1	320	62	100
2	127	2.2	11
3	112	11	32
4	42	0.6	6.4
5	520	8.7	24
6	190	7.2	16
7	210	2.2	6.4
8	49	9.2	16
9	2000	18	79
10	140	4.7	59
11	180	7.4	100
12	120	5.5	23
13	166	1.9	6.3
14	67	2.6	19
15	65	2.6	16
16	80	3.3	22
17	140	3.2	19
18	210	10	30
19	48	1.8	10
20	270	7.2	46
21	217	8.9	>100
22	47	2.1	5.2
23	86	7.9	24
24	207	5.6	42

Table S2. Bovine thyroglobulin immunoreactive peptides identified by MS/MS analysis from 1D PAGE with high confidence

Peptide	Sequence	#PSMs	XCorr	Charge	MH+ [Da]	RT [min]
Mass range 15 – 20 kDa						
1	$_{2485}$VDLLIGSSQDDGLINR$_{2501}$	6	1.66	3	1715.8835	23.68
2	$_{2244}$ARCWQPGIR$_{2252}$	1	0.79	2	1144.5590	20.04
Mass range 10 – 15 kDa						
3	$_{2291}$GSGDRPAVDGSFLAAVGNLIVVTASYR$_{2319}$	1	4.15	3	2692.4073	39.17
4	$_{2131}$CLWECSR$_{2137}$	1	1.65	2	1010.4180	27.36
5	$_{2129}$DRCLWECSR$_{2137}$	1	1.54	2	1281.5469	26.38
6	$_{2020}$GGEVTCLTLNSLGLQTCSEEYGGVWR$_{2045}$	2	8.51	3	2886.3376	35.44
7	$_{1981}$NKVPMSDKSISSGFFECER$_{1999}$	2	3.01	4	2218.0257	28.64
8	$_{1988}$SISSGFFECER$_{1999}$	1	2.95	2	1318.5736	30.23
9	$_{1647}$SEDALGTSQATSFGSLQCQVK$_{1667}$	2	6.83	2	2214.0349	30.75
10	$_{1581}$VIFSADVAVMVR$_{1592}$	1	3.98	2	1306.7199	33.22
11	$_{1379}$FADLIQSGTFQLHLDSK$_{1395}$	1	3.54	3	1919.9877	33.44
12	$_{1337}$TAGTPVSIPVCDDSSVKVECLSR$_{1359}$	6	3.27	3	2477.2020	30.42
13	$_{1309}$GFCQIQVK$_{1316}$	1	1.96	2	979.5027	26.86
14	$_{1305}$VCSADYSGLLLAFQVFLLDELTAR$_{1328}$	2	6.48	3	2701.3937	44.39
15	$_{1255}$SAFPPEPLLCSVQR$_{1268}$	4	1.95	2	1600.8146	33.36
16	$_{1171}$AEDGGFSPVQCDPAQGSCWCVLGSGEEVPGTR$_{1202}$	1	7.03	3	3409.4571	33.82
17	$_{996}$LAAQSTFDFYQR$_{1007}$	1	1.41	2	1446.7013	31.06
18	$_{414}$ELFLDSGIFQPMLQGR$_{429}$	2	1.61	3	1851.9279	31.19
19	$_{284}$FLAVQLVISGR$_{294}$	1	2.59	2	1202.7255	34.01
20	$_{214}$FPDAFVTFSSFR$_{228}$	1	3.01	2	1420.6903	35.59
21	$_{179}$SPPQCSPDGAFRPVQCK$_{195}$	1	1.67	3	1930.8890	25.66
22	$_{171}$LLHGVGDRSPPQCSPDGAFRPVQCK$_{195}$	1	1.08	5	2778.3555	25.56
23	$_{85}$QPGRPAACLSFCQLQK$_{100}$	1	0.57	3	1862.8807	25.36
